# Detection of bacterial antigens and Alzheimer’s disease-like pathology in the central nervous system of BALB/c mice following intranasal infection with a laboratory isolate of *Chlamydia pneumoniae*

**DOI:** 10.3389/fnagi.2014.00304

**Published:** 2014-12-05

**Authors:** Christopher S. Little, Timothy A. Joyce, Christine J. Hammond, Hazem Matta, David Cahn, Denah M. Appelt, Brian J. Balin

**Affiliations:** ^1^Department of Bio-Medical Sciences, Philadelphia College of Osteopathic MedicinePhiladelphia, PA USA; ^2^Center for Chronic Disorders of Aging, Philadelphia College of Osteopathic MedicinePhiladelphia, PA, USA; ^3^Division of Research, Philadelphia College of Osteopathic MedicinePhiladelphia, PA, USA

**Keywords:** Alzheimer, infection, *Chlamydia pneumoniae*, amyloid beta, bacteria

## Abstract

Pathology consistent with that observed in Alzheimer’s disease (AD) has previously been documented following intranasal infection of normal wild-type mice with *Chlamydia pneumoniae* (Cpn) isolated from an AD brain (96-41). In the current study, BALB/c mice were intranasally infected with a laboratory strain of Cpn, AR-39, and brain and olfactory bulbs were obtained at 1–4 months post-infection (pi). Immunohistochemistry for amyloid beta or Cpn antigens was performed on sections from brains of infected or mock-infected mice. *Chlamydia*-specific immunolabeling was identified in olfactory bulb tissues and in cerebrum of AR-39 infected mice. The Cpn specific labeling was most prominent at 1 month pi and the greatest burden of amyloid deposition was noted at 2 months pi, whereas both decreased at 3 and 4 months. Viable Cpn was recovered from olfactory bulbs of 3 of 3 experimentally infected mice at 1 and 3 months pi, and in 2 of 3 mice at 4 months pi. In contrast, in cortical tissues of infected mice at 1 and 4 months pi no viable organism was obtained. At 3 months pi, only 1 of 3 mice had a measurable burden of viable Cpn from the cortical tissues. Mock-infected mice (0 of 3) had no detectable Cpn in either olfactory bulbs or cortical tissues. These data indicate that the AR-39 isolate of Cpn establishes a limited infection predominantly in the olfactory bulbs of BALB/c mice. Although infection with the laboratory strain of Cpn promotes deposition of amyloid beta, this appears to resolve following reduction of the Cpn antigen burden over time. Our data suggest that infection with the AR-39 laboratory isolate of Cpn results in a different course of amyloid beta deposition and ultimate resolution than that observed following infection with the human AD-brain Cpn isolate, 96-41. These data further support that there may be differences, possibly in virulence factors, between Cpn isolates in the generation of sustainable AD pathology.

## INTRODUCTION

Alzheimer’s disease (AD) is the most common dementia in the US, accounting for 50–70% of cases. More than 5 million Americans are living with a diagnosis of AD as of 2013 with 90–95% of cases in the 65 and older segment of the population. Early stage of disease involves memory impairment (Fargo and Bleiler, 2014). In the advanced stages of AD, individuals require assistance with daily activities and, ultimately, in the final stage become bed-bound and are reliant on around-the-clock care (Hebert et al., 2003). AD is a fatal disorder with the progression from the earliest symptoms to total functional dependency and death in an untreated person often occurring within 8–10 years post-diagnosis (Fargo and Bleiler, 2014).

Although much is known about the disease process and progression of AD, the initiating factors or cause(s) of the disease still remain a mystery. AD has an early onset or “familial form” that is an autosomal dominant disorder, primarily driven by genetic alterations in genes encoding the beta amyloid precursor protein or the loci encoding the enzymes that process this precursor, presenilins 1 and 2 (Goate et al., 1991; Levy-Lahad et al., 1995; Rogaev et al., 1995; Wolfe, 2007). Transgenic mouse models have been developed with enhanced β-amyloid production and deposition (Wisniewski and Sigurdsson, 2010; Hall and Roberson, 2012), and serve as models for the “early onset” familial form of AD, which accounts for 5% or fewer of all reported cases. One deficiency of these model systems is how to target the early initiating events in sporadic late-onset AD and not just the “tombstone” lesions that are the result of years or decades of progressive pathological processes (Wisniewski and Sigurdsson, 2010). In this regard, animal models that mimic aspects of the sporadic late-onset form of AD have been developed, but lack a clear understanding of the primary factors promoting β-amyloid deposition. Models that have been used to experimentally induce AD-like pathology in the central nervous system (CNS) have focused on chronic stress (Alkadhi et al., 2010), chemical induction with colchicine (Kumar et al., 2007), and bacterial toxins such as streptozotocin (Labak et al., 2010; for review see Balin et al., 2011). A limited number of infectious agents, including *Chlamydia pneumoniae* (Cpn), have been proposed to enhance risk or play a contributing or causal role in AD (Balin et al., 1998; Gerard et al., 2006); animal models have been developed to study the effects of this infection (Little et al., 2004, 2005) with regards to AD-like pathology. However, there remains a dearth of experimental animal systems that accurately model the initiation and progression of sporadic/late-onset AD, leaving researchers with limited options to address pertinent questions pertaining to these important aspects of this chronic disease.

The identification of Cpn in AD brain tissue (Balin et al., 1998) was the impetus to investigate the potential role of infection, with this obligate intracellular bacterium, in the induction and progression of late-onset AD and led to the establishment of a mouse model to investigate this occurrence (Little et al., 2004). In the original experimental system, BALB/c mice were infected with Cpn recovered from AD brain tissue. The isolate of Cpn, 96-41, was briefly propagated in an epithelial cell line and then used to infect 3 months old BALB/c mice intranasally; brain tissue was analyzed for AD-like pathology at monthly intervals up through 3 months pi following infection in this manner.

This initial study utilized the human AD-brain isolate of Cpn to evaluate whether AD-like pathology was an outcome in non-transgenic mice (Little et al., 2004), and was designed to address Koch’s postulates. To fulfill the first postulate, the infectious organism must be isolated from tissues of an affected individual. In this particular case, the first postulate was satisfied, but for other cases this issue is still debated (Itzhaki et al., 2004). Koch’s second postulate dictates that the pathogen be isolated from a diseased organism followed by growth in pure culture. The bacterium was isolated post-mortem from AD-brain tissue and grown in culture within a host cell as this is an obligate intracellular bacterium. Third, the organism was introduced into a mouse via the natural route of infection, and induced pathology consistent with AD, while mice receiving vehicle alone did not display the same pathology. Koch’s fourth postulate stipulates that the organism be re-isolated from affected animals; in this instance, Cpn was identified in the tissues of affected mice, but was not re-isolated from the tissue (Balin et al., 2011). Koch’s postulates were used as a general guide, and when studying an intracellular infection these observations are consistent with the hypothesis that Cpn infection can induce AD-like pathology, specifically β-amyloid deposition, in the brain and contribute directly to pathogenesis.

In mice infected with Cpn, a detectable difference in β-amyloid deposits was observed at 2 months pi, and a greater number of deposits were identified at 3 months pi. The increase in both the number and size of amyloid deposits at later timepoints indicated that there was progressive development of AD-like pathology. The experimental induction of BALB/c mouse derived β-amyloid deposits at 5 and 6 months of age (2 and 3 months pi) also suggests that infection was directly responsible for the production and deposition of this β-amyloid. In contrast, in transgenic mouse models used to study AD, substantial amyloid deposits are not typically found at 3 or even 6 months of age, yet substantial pathology was induced within 3 months following the introduction of the infectious agent into this non-transgenic mouse model of sporadic AD (Little et al., 2004). As Cpn is typically associated with an acute respiratory illness, introduction into BALB/c mice was via intranasal inoculation, the natural route of infection. Additional experimental evidence supports the hypothesis that the respiratory infection precedes dissemination to other organ systems (Little et al., 2005). In this regard, while spread of the organism occurs in younger animals, it is even more apparent with the advent of immunosenescence in older animals.

In contrast with the initial report associating Cpn with the induction of AD-like pathology in the brains of BALB/c mice (Little et al., 2004), the current study was performed with a respiratory isolate and common laboratory strain of Cpn, AR-39. The purpose was to determine if this well-studied laboratory isolate of Cpn would induce pathology in a similar manner and to the same degree over a similar time course, as that observed for the human CNS isolate used previously. This approach will inform potential differences in outcomes when infecting mice with Cpn originally isolated from lung tissues and used as a laboratory isolate as compared to that from human AD brain.

## MATERIALS AND METHODS

### HEp-2 CELL LINE

The human epithelial, HEp-2, cell line (ATCC, Rockville, MD, USA) was cultured in minimal essential medium (MEM) supplemented with 10% fetal bovine serum (FBS; Cellgro Mediatech, Inc., Manassas, VA, USA), 5 mM L-Glutamine (Thermo Fisher Scientific, Pittsburgh, PA, USA) at 37°C and 5% CO_2_. 1–2 × 10^5^ cells were plated in a T25 tissue culture flask (Thermo Fisher Scientific, Pittsburgh, PA, USA) and passaged as needed prior to collection for the propagation of Cpn.

### PROPAGATION AND PURIFICATION OF *Chlamydia pneumoniae*

*Chlamydia pneumoniae*, AR-39 isolate, was obtained from the ATCC (ATCC, Rockville, MD, USA) and propagated in the HEp-2 cell line similar to that described for the Cpn brain isolate, 96-41 (Campbell et al., 1991; Little et al., 2004). Prior to infection of BALB/c mice, homogenates of 72 h culture supernatants and Cpn infected HEp-2 cells were sonicated for 30 s and passed through a series of filter membranes with decreasing pore size to collect the elementary bodies. The organism was resuspended in Hanks balanced salt solution (HBSS), aliquoted, and stored at –80°C. The quantification of inclusion forming units (IFUs) subsequently was determined following infection of HEp-2 epithelial cells with a series of 10-fold serial dilutions of the concentrated organism. The inclusions were identified by immunofluorescence using a *Chlamydia*-specific antibody (Imagen^TM^; DAKO, Carpenteria, CA, USA). Immediately preceding infection, aliquots were diluted in HBSS for the intranasal infection of mice.

### MICE

Six week old female BALB/cJ mice were purchased from Jackson Laboratories (Bar Harbor, ME, USA) and acclimated for 2 weeks prior to the initiation of experiments. Mice were housed in groups of 2–3 in HEPA-filter caged racks, with infected mice housed separately from uninfected mice, within the bio-containment facility at the Philadelphia College of Osteopathic Medicine. All animal husbandry was performed using Biosafety Level 2 precautions and in a Class II biosafety cabinet. Mice were fed food and water ad libitum. All animal protocols were approved by the IACUC at PCOM.

### INFECTION OF MICE WITH *Chlamydia pneumoniae*

Under manual restraint, 8 week old, female BALB/cJ mice were inoculated intranasally with 5 × 10^5^ IFUs of the AR-39 isolate of Cpn diluted in 50 μl of HBSS. Six mice were inoculated at 8 weeks of age for each time point and the brains were collected at 1, 2, 3, and 4 months post-infection (pi) for analysis. Four age and sex matched mice were mock-intranasally infected with vehicle alone, HBSS, as a control for each time point. At each time point, three experimentally infected and two mock-infected control mice were anesthetized, cardiac-perfused and organs were collected and immersion fixed in 4% paraformaldehyde for embedding, sectioning, and immunohistochemical analysis. The remaining three experimentally infected and two mock-infected control mice at each time point other than for 2 month animals for which frozen tissue was not available were euthanized and organs were collected and snap-frozen in liquid nitrogen and then stored at –80°C until analysis for detection and quantification of viable organism.

### RECOVERY AND QUANTIFICATION OF *Chlamydia pneumoniae*

Quantification of viable Cpn was performed in an identical manner to our previous report (Little et al., 2005) in the following manner. Frozen tissue was thawed and a 10% weight to volume homogenate was prepared in serum-free MEM (Thermo Fisher Scientific, Pittsburgh, PA, USA) supplemented with 2 mM Glutamine. Serial 10-fold dilutions (in 200 μL) were added to four well Lab Tech chamber slides (Naperville, IL, USA) on which HEp-2 cells were previously plated. Negative control wells contained cells mock-infected with medium alone. Chamber slides were incubated at 37°C in 5% CO_2_ for 2.5 h, washed with HBSS and refilled with fresh complete medium supplemented with 2 μg/ml cycloheximide (Sigma-Aldrich, St. Louis, MO, USA) followed by incubation for 48 h at 37°C. After incubation, slides were washed with HBSS, fixed in 50% methanol at RT for 20 min, washed twice in HBSS, and labeled with a 1:10 dilution of FITC-conjugated *Chlamydia*-specific antibody (Imagen^TM^; DAKO, Carpenteria, CA, USA) for 90 min at 37°C, protected from light. Slides were washed in phosphate buffered saline (PBS) and counterstained with a 2 μg/ml of Bisbenzamide (Sigma-Aldrich, St. Louis, MO, USA) in PBS for 1 min, washed in PBS and coverslipped with aqueous mounting medium (Imagen^TM^; DAKO, Carpenteria, CA, USA). All titers are calculated as IFUs/ml of 10% weight to volume tissue homogenate.

### ANTIBODIES

The following *Chlamydia*-specific antibodies were generated in mice: RDI-PROAC1p (Research Diagnostics Incorporated, Flanders, NJ, USA; AC1P; monoclonal I_g_G) specific for *Chlamydia* lipopolysaccharide used at a dilution of 1:10 (5 μg/ml), M6600 (DakoCytomation, Carpinteria, CA, USA; monoclonal I_g_G) specific for Cpn major outer membrane protein used at a dilution of 1:10 (10 μg/ml), and 10C-27 (Fitzgerald, Concord, MA, USA; monoclonal I_g_G) specific for Cpn used at a dilution of 1:100 (1 μg/ml). Additionally, B65256R (Biodesign International, Saco, ME, USA; B56R) specific for *Chlamydia* purified elementary bodies was generated in rabbit and used at a dilution of 1:200 (2 μg/ml). Both secondary antibodies specific for either mouse, AP-Goat anti-mouse IgG conjugate (Zymed Laboratories, San Francisco, CA, USA), or rabbit, AP-Goat anti-rabbit IgG conjugate (Zymed Laboratories, San Francisco, CA, USA), were used at a concentration of 2 μg/ml. All antibodies were diluted to working concentration in 2% FBS/PBS blocking buffer (Thermo Fisher Scientific, Pittsburgh, PA, USA). For the detection of Aβ-amyloid, the following antibodies were used at a recommended concentration of 2 μg/ml: a rabbit polyclonal antibody specific for the carboxyl-terminal fragment of Aβ amyloid 1–42 (catalog: A1976 Oncogene Research Products, Boston, MA, USA), and a mouse monoclonal antibody (4G8) to the 17–24 amino acid peptide of human Aβ amyloid 1–42 (catalog:9220-05 Signet Laboratories Inc., Dedham, MA, USA). For all amyloid-specific immunolabeling, secondary antibodies consisted of HRP conjugated sheep anti-Mouse IgG (H + L) or donkey anti-rabbit IgG (H + L). Antibodies were used at a dilution of 1:300 as recommended by the supplier (Amersham Biosciences, Piscataway, NJ, USA and Life Technologies, Inc., Grand Island, NY, USA).

### IMMUNOHISTOCHEMISTRY

Brain sections from experimental and control mice were immunolabeled for Aβ-amyloid or Cpn antigen at 1, 2, 3, and 4 months pi using the aforementioned antibodies. Coronal sections were deparaffinized with xylene (Thermo Fisher Scientific, Pittsburgh, PA, USA) rehydrated in a series of graded alcohol solutions (Electron Microscopy Sciences, Fort Washington, PA, USA), followed by de-ionized (DI) H_2_O. Slides were then placed in Citra antigen retrieval buffer (BioGenex, San Ramon, CA, USA) and steamed in a 2100 Retriever (Pick Cell Laboratories, Amsterdam, Netherlands) for 20 min at high pressure and temperature (120°C). Slides were then rinsed with PBS pH 7.4 (Sigma-Aldrich, St Louis, MO, USA) 3 × 5 min. Endogenous peroxidase activity was quenched utilizing a 3% solution of H_2_O_2_/PBS (Thermo Fisher Scientific, Pittsburgh, PA, USA) for 5 min at RT. Sections were rinsed 1 × 5 min in PBS and blocked 3 × 15 min in 2% heat inactivated FBS/PBS. A total of 30 coronal brain sections, 10 sets of three sections (one per antibody), were immunolabeled per mouse. The sections were spaced equally (approximately every 70–100 microns in brain tissue) from rostral (bregma +2.22 mm) to caudal (bregma –5.88 mm) in order to provide samples representative of the regions spanning the entire brain of each mouse. Slides receiving *Chlamydia*-specific primary antibodies B56R or a cocktail of 10C-27, AC1P, M6600 were applied to tissue sections and placed in a humidified chamber at 37°C for 90 min. The sections were rinsed 3 × 5 min each and then blocked 3 × 15 min each in 2% FBS/PBS, and incubated with appropriate secondary antibodies for 1 h at 37°C. Next, sections were rinsed with DI H_2_O 3 × 5 min and developed using alkaline phosphatase new magenta for 15 min (BioFX, Owings Mills, MD, USA) at RT. Sections were rinsed in DI H_2_O 3 × 5 min followed by one PBS rinse for 5 min. Acidified Harris’s Hematoxylin (Thermo Fisher Scientific, Pittsburgh, PA, USA) was applied to sections for 1 min. One DI H_2_O rinse followed counterstaining and the sections were contrasted in PBS for 5 min. Finally, the sections were rinsed with DI H_2_O 3 × 5 min, air dried, and crystal mounted (BioMeda, Foster City, CA, USA). Once dry, the sections were permounted and coverslipped.

Slides receiving mouse primary antibodies were blocked in mouse on mouse (M.O.M.) IgG blocking reagent (Vector M.O.M. kit, Vector Laboratories, Burlingame, CA, USA) for 60 min at RT, rinsed, and incubated for 5 min in the M.O.M. blocking buffer. Following blocking, primary antibodies were added to samples and incubated overnight at 4°C. The sections were rinsed in PBS 3x for 5 min each, blocked 3x for 15 min each in 2% FBS/PBS, and incubated with appropriate secondary antibodies for 120 min at RT. The sections labeled with anti-amyloid antibodies were rinsed with PBS 3x for 10 min each and visualized with 3, 3’-Diaminobenzidine (DAB; Sigma *FAST*^TM^, Sigma-Aldrich, St. Louis, MO, USA). Sections were rinsed with dH_2_O, counterstained with Harris’ Alum Hematoxylin for 1 min (EM Sciences Harleco^®^, EM Industries, Inc., Hawthorne, NY, USA), dehydrated and permounted.

### MICROSCOPIC ANALYSIS

Digital images were captured using Image-Pro Plus Phase 3 Imaging System software (Media Cybernetics, Silver Spring, MD, USA) on a Nikon Eclipse E800 microscope using a Spot RT Camera (Diagnostic Instruments, Sterling Heights, MI, USA).

### STATISTICAL ANALYSIS

Statistical analysis was performed using the student *t*-test followed by pair-wise testing of uninfected (*n* = 8) relative to each experimental infected timepoint (*n* = 3) using Microsoft excel statistical analysis software and *P* values of <0.05 which were considered significant.

## RESULTS

### RECOVERY OF INFECTIOUS *Chlamydia pneumoniae* FROM OLFACTORY BULBS AND CEREBRUM

**FIGURE 1:**
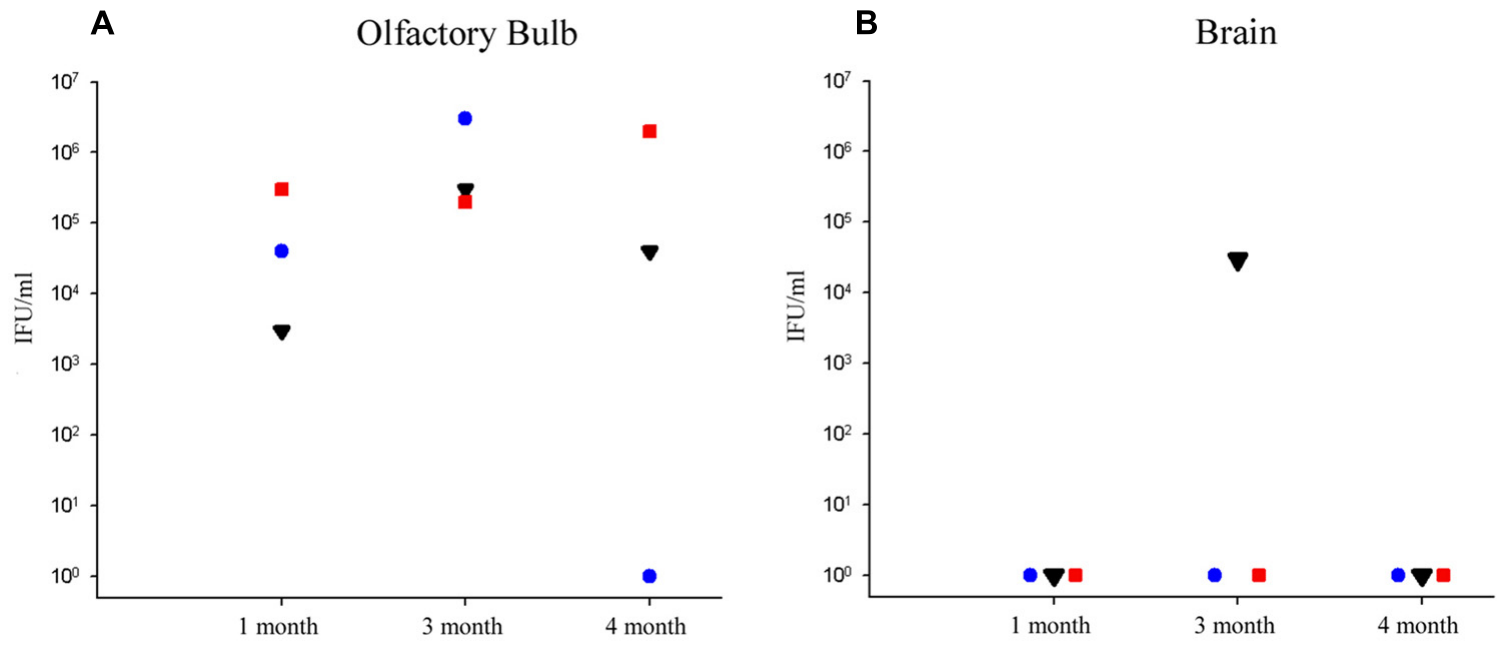
**Recovery of viable *Chlamydia pneumoniae* from olfactory bulb and brain tissues following intranasal infection. (A)** At 1, 3, and 4 months post-infection (pi), viable Cpn was recovered from olfactory bulb tissue homogenates of eight BALB/c mice, 3 of 3 mice at 1 month pi, 3 of 3 mice at 3 months pi, and 2 of 3 mice at 4 months pi. **(B)** In contrast, only 1 mouse demonstrated viable Cpn from cerebral cortical tissue at any time; that being one mouse at 3 months pi. Viable Cpn was quantified as infectious forming units/ml of tissue homogenate.

Olfactory bulbs and cerebral tissues were dissected from BALB/c mice following euthanization, snap-frozen, and homogenized prior to incubation with HEp-2 cells in culture to determine if detectable levels of viable Cpn could be recovered from the CNS. Ten-fold serial dilutions of the homogenized tissues were incubated with HEp-2 cells to determine the amount of viable infectious Cpn present in the tissues at 1, 3, and 4 months pi. Tissue from the 2 month animal was not available. Infectious Cpn was recovered and quantified from 3 of 3 olfactory bulbs at 1 month pi, ranging from 3 × 10^3^ to 3 × 10^5^ IFU/ml of tissue homogenate (**Figure 1A**). At 3 months pi, Cpn was detected in 3 of 3 olfactory bulbs with a range of 2 × 10^5^ to 3 × 10^6^ IFU/ml of tissue homogenate (**Figure 1A**). At 4 months, Cpn was detected in 2 of 3 olfactory bulbs with a range of 0 to 2 × 10^6^ IFU/ml tissue homogenate (**Figure 1A**). Of the three olfactory bulbs tested from the mock-infected animals, no Cpn was recovered. In contrast to the olfactory bulbs, Cpn was not recovered from the brain tissue (cerebrum) at 1 and 4 months pi, although at 3 months, Cpn was recovered and quantified at 3 × 10^4^ IFU/ml of tissue from 1 of 3 brains (**Figure 1B**). This same mouse had 3 × 10^5^ IFU/ml in the olfactory bulb as noted above. With regards to brain tissues analyzed from the three control animals, no Cpn was detected.

### IDENTIFICATION AND DISTRIBUTION OF *Chlamydia pneumoniae* ANTIGEN IN THE CENTRAL NERVOUS SYSTEM

**FIGURE 2:**
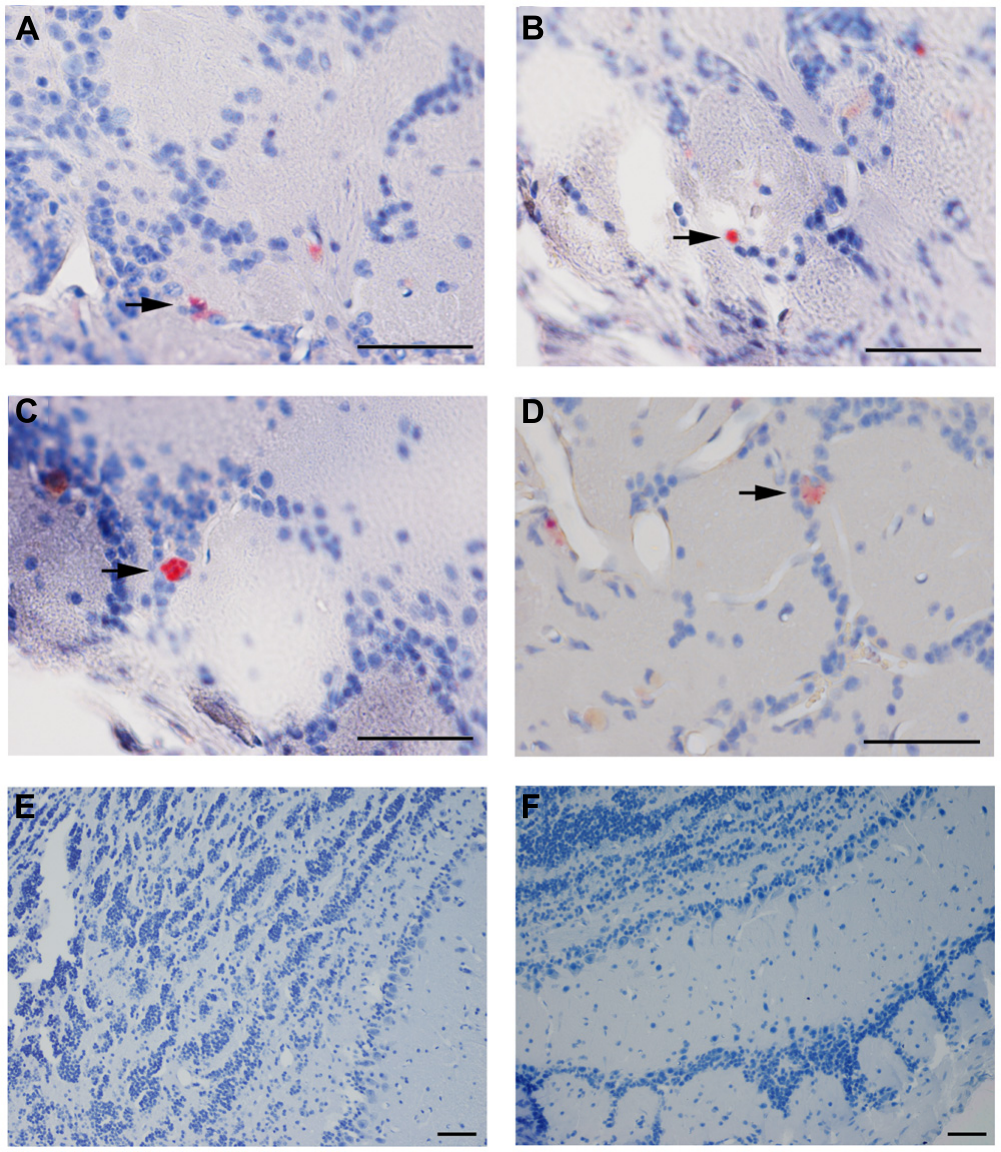
***Chlamydia pneumoniae* specific immunoreactivity in olfactory bulbs.** Cpn (AR-39) antigens were detected in olfactory bulb tissues at 1 month pi following intranasal inoculation (arrows). **(A,B)** are from 1 infected mouse and **(C,D)** from a separate mouse infected with Cpn and labeled with a cocktail of anti-Cpn antibodies (RDI-PROAC1p, M6600, 10C-27). **(E,F)** are representative images from mock-infected mice comparably immunolabeled. Mag bars **(A–F)** = 50 μm.

**FIGURE 3:**
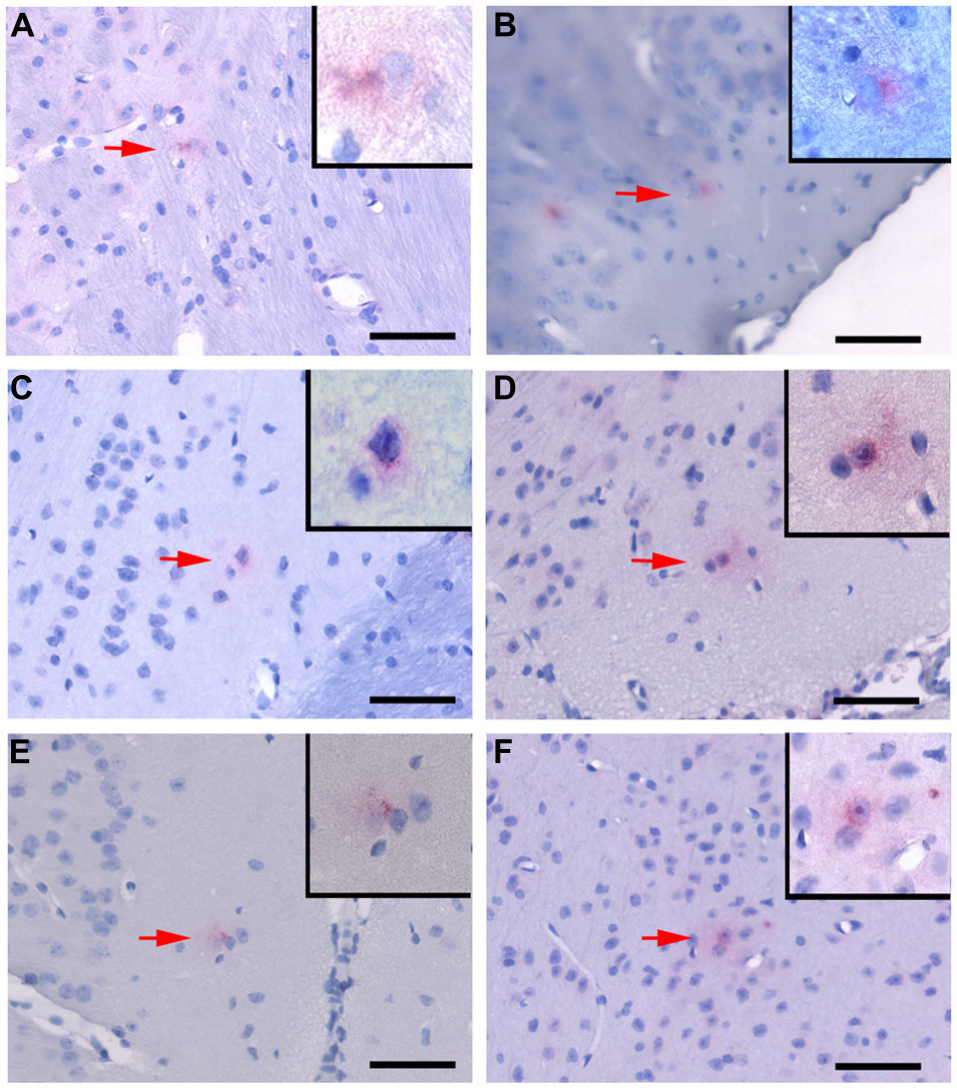
***Chlamydia pneumoniae* specific immunoreactivity in the central nervous system.** Representative images of Cpn-specific antigen labeling in the brains of infected mice at 1 month **(A,B)**, 3 months **(C,D)**, and 4 months **(E,F)** pi. The upper right corner of each image is a higher magnification image of Cpn-specific antigen labeling as designated by the low magnification arrow. Mag bars = 50 μm.

Cpn antigen was detected in olfactory bulb tissues at 1 and 3 months pi using antibodies specific for Cpn LPS and outer membrane proteins. Representative immunolabeling for Cpn in these tissues at 1 month pi was principally intracellular (**Figure 2**). The labeling profiles consisted of large intracellular vacuoles, often perinuclear with prominent well-defined inclusions. Furthermore, Cpn antigen labeling (LPS and outer membrane proteins) was documented within the cerebrum with a quantitative analysis of 10 total slides per animal distributed rostral to caudal with distances measured from bregma (**Table 1**). Intracellular immunolabeling was observed to be both perinuclear and diffuse in the cytoplasm with very few clearly documentable intracellular inclusions (**Figure 3**). However, upon close examination, punctate immunolabeling was observed in numerous cells (**Figures 3C,E**).

**Table 1:** Location of *Chlamydia pneumoniae* immunoreactivity and Aβ 1–42 amyloid deposits over 4 months pi within brains of Cpn*-*infected mice.

A
	Bregma									Total	
	2.22	1.7	0.38	–1.28	–2.75	–3.8	–4.92	–5.46	–5.88	Inf	Un
1 Mo Cpn	22	10	22	18	23	42	9	6	2	154	24
Amyloid	0	1	0	3	3	0	2	1	0	10	0
2 Mo Cpn	18	15	19	5	26	21	20	10	0	134	14
Amyloid	8	30	11	16	31	43	35	6	0	180	12
3 Mo Cpn	11	11	13	22	5	9	9	4	7	91	10
Amyloid	0	9	14	7	4	3	3	11	2	53	10
4 Mo Cpn	0	11	13	15	13	10	10	1	3	76	9
Amyloid	0	0	3	6	3	1	5	1	0	19	5

Cpn	51	47	67	60	67	82	48	21	12	455	57
Amyloid	8	33	12	5	32	19	33	1	0	143	27

**B**
	*Chlamydia-specific immunoreactivity*					**Amyloid deposits**			
	**Uninfected (*n* = 2)**		**Infected (*n* = 3)**		****	**Uninfected (*n* = 2)**		**Infected (*n* = 3)**	
**Time pi**	**mean**	**SD**	**Mean**	**SD**	**Time pi**	**Mean**	**SD**	**Mean**	**SD**

1	12	5.65	51.33*	32.01	1	0	0	3.33	1.167
2	7	2.83	44.67*	33.56	2	6	1	60*	8
3	5	1.41	30.33*	12.06	3	5	1	17.67*	0.67
4	4.50	0.71	25.33	24.21	4	2.5	1.5	6.33	2.167
ALL	7.13	4.02	37.92	21.22	ALL	3.38	2.28	21.83	19.08

**(A)** The location and number of immunoreactive amyloid deposits or Cpn antigen is designated in millimeters (section location in mm) rostral or caudal to the mouse bregma. **(B)** Statistical analysis of Cpn-specific immunoreactivity and Aâ 1–42 immunoreactive deposits from infected and uninfected mouse brains. For each time point, n = animals analyzed, *indicate statistical significance.

Quantitative analysis of Cpn antigen in the brain at 1 through 4 months post-intranasal inoculation revealed peak Cpn antigen burden (154 immunoreactive profiles) at 1 month (**Table 1A**). Cpn-specific immunoreactivity demonstrated a step-wise decrease at 2, 3, and 4 months pi with 134, 91, and 76 immunoreactive profiles, respectively. With regards to specific coordinates in the brain, the greatest Cpn antigen burden (i.e., 42 Cpn immunoreactive profiles) was documented at 1 month in multiple sections 3.8 mm caudal to bregma. These sections contain the entorhinal cortex (Ect), perirhinal cortex (Prh), hippocampus, and amygdala, all regions affected in AD. A low but detectable number of non-specific Cpn immunoreactive sites were detected within mock-infected control mouse brain tissue with an average number of 0.355/section analyzed. The mean number of immunoreactive sites identified was 7.125/mouse ± 4.01 and based upon the results of the student *t*-test a statistically significant difference (*p* < 0.05) was observed between experimentally infected tissue and mock-infected control mouse tissue at all timepoints analyzed; 1 month pi (51.33 ± 32.01), 2 months pi (44.67 ± 33.56), and 3 months pi (30.33 ± 12.06). No statistically significant difference was observed in the 4 month pi (25.33 ± 24.21) experimental group relative to uninfected control tissue (**Table 1B**).

### IDENTIFICATION AND DISTRIBUTION OF AMYLOID ANTIGEN IN THE CENTRAL NERVOUS SYSTEM

Antibodies specific for amyloid beta 1–40 (Aβ 1–40) and amyloid beta 1–42 (Aβ 1–42) were used to determine if immunoreactive deposits could be detected in mock-infected controls and experimentally infected BALB/c mice. A limited number of Aβ 1–40 immunoreactive deposits were observed exclusively in the brains of experimentally infected mice at 2 months pi (data not shown). No Aβ 1–40 deposits were detected in the brains of any control mice at any timepoint nor in experimentally infected mice at 1, 3, and 4 months pi.

**FIGURE 4:**
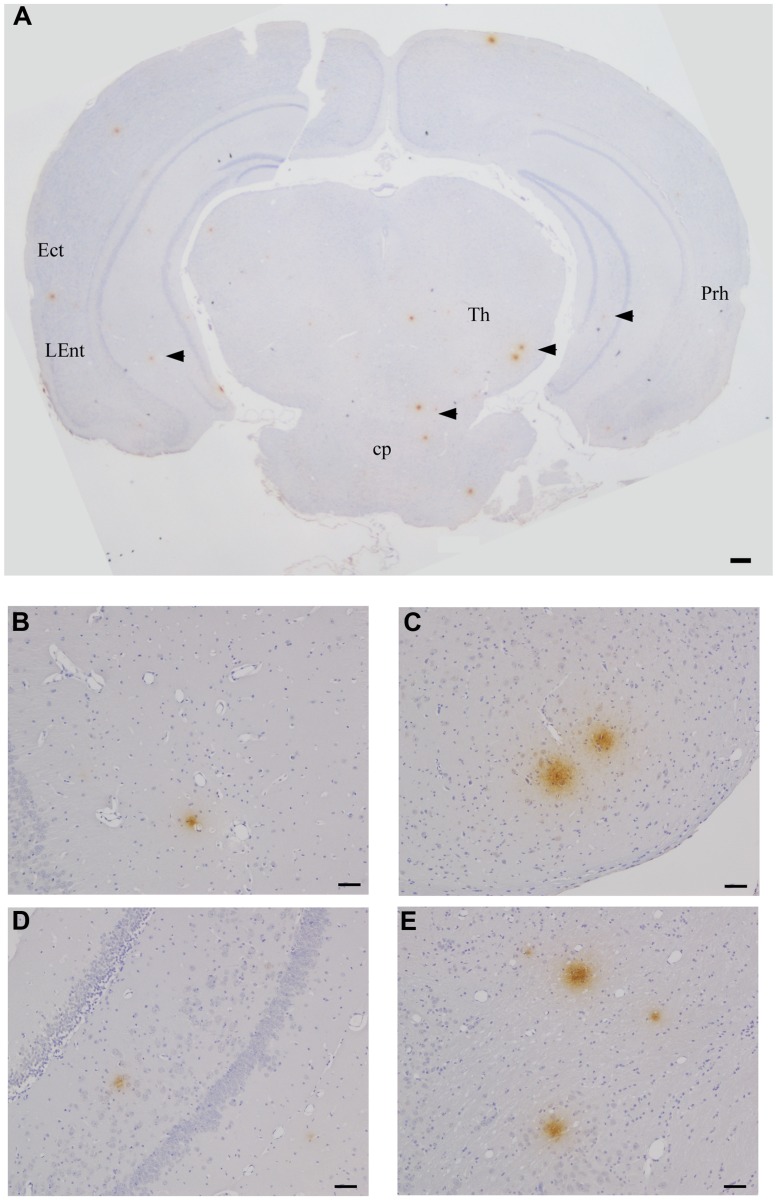
**Beta amyloid Aβ 1–42 deposits in the CNS at 2 months pi following intranasal infection with *Chlamydia pneumoniae* AR-39.** Brains were examined by light microscopy for the presence of Aβ 1–42 using a specific anti-Aβ 1–42 antibody. **(A–E)** Representative images of Aβ 1–42-specific labeling (arrowheads) are shown within different regions of this brain section. Ect (entorhinal cortex), L Ent (lateral entorhinal cortex), Th (thalamus), Prh (perirhinal cortex), Cp (cerebral peduncle). Mag bars **(A)** = 100μm; **(B–E)** = 20 μm.

Quantitative analysis of amyloid burden revealed the highest number of Aβ 1–42 immunoreactive deposits (43) at 2 months pi 3.8 mm caudal to bregma, similar to Cpn immunoreactivity at 1 month pi (**Table 1A**). Overall Aβ 1–42 immunoreactivity was greatest at 2 months pi, having been minimal at 1 month pi and decreasing at 3 and 4 months pi. As noted above, these sections contain the Ect, Prh, cerebral peduncle (Cp), hippocampus, and amygdala, all regions affected in AD (see **Figure 4** for Aβ 1–42 immunoreactive deposits). A low but detectable number of amyloid-specific immunoreactive sites were detected within mock-infected control mouse brain tissue at 2, 3, and 4 months pi with an average number of 0.17/section analyzed. The mean number of immunoreactive sites identified was 3.38/mouse ± 2.28 and based upon the results of the student *t*-test a statistically significant difference (*p* < 0.05) was observed between experimentally infected tissue and mock-infected control mouse tissue at 2 months pi (60/mouse ± 8) and 3 months pi (17.67 ± 0.67). No statistically significant difference was detected at 1 month pi (3.33 ± 1.17) or 4 months pi (21.83 ± 19.08; **Table 1B**).

## DISCUSSION

This study was designed as a follow-up investigation to the initial report of experimental induction of AD-like pathology in BALB/c mice following intranasal inoculation with *C. pneumoniae* (Little et al., 2004). The key difference in the current study as compared to that by Little et al. (2004) was that the AR39 respiratory lab strain was used to evaluate the effects in the brain as compared to the 96-41 brain strain used in the initial report. *Chlamydia*-specific immunolabeling was identified in olfactory bulb tissues and in brains (cortical tissues) of AR-39-infected mice. The Cpn-specific labeling was most prominent at 1 month pi and the greatest burden of amyloid deposition was noted at 2 months pi, whereas both decreased at 3 and 4 months pi. The majority of amyloid deposits at these times were immunoreactive for Aβ 1–42. Interestingly, a limited number of Aβ 1–40 immunoreactive deposits also was identified (data not shown), but only at the 2 month time point, the time of peak amyloid burden. Viable Cpn was recovered from the olfactory bulb tissues of 3 of 3 experimentally infected mice at 1 and 3 months pi, and 2 of 3 at 4 months pi. In contrast, in cerebral cortical tissues of experimentally infected mice, only at 3 months pi did 1 of 3 mice have a measurable burden of viable Cpn. Mock-infected control mice had no detectable Cpn in either olfactory bulbs (0 of 3) or cortical tissues (0 of 3). These data indicate that, following intranasal infection, the AR-39 respiratory isolate of Cpn establishes a limited infection predominantly in the olfactory bulbs of BALB/c mice. Furthermore, although infection with the laboratory strain of Cpn promotes deposition of Aβ-amyloid, this appears to resolve following reduction of the Cpn antigen burden over time.

In our current study, brains were analyzed at 1 through 4 months pi by immunohistochemistry using specific antibodies for both *Chlamydia* antigen and Aβ-amyloid 1–42. Similar to our initial report utilizing the AD-brain isolate 96-41, no substantial amyloid deposits were observed at 1 month pi with AR-39 and only a limited degree of pathology was identified at 2 months pi. In contrast to the original study using the brain isolate, at 4 months pi, AD-like pathology was comparable to that observed in mock-infected mice as well as infected mice at 1 month pi, suggesting a decrease of pathology between 2 through 4 months pi. Identification and quantitative analysis of the *Chlamydia* burden indicated that peak antigen burden preceded the peak deposition of amyloid. The greatest *Chlamydia* antigen burden in infected BALB/c mice was noted at 1 month pi, and decreased at 2 through 4 months pi, whereas peak amyloid burden was at 2 months pi, and decreased thereafter. Taken together, the relationship between *Chlamydia* antigen burden and the number of amyloid deposits suggests that Cpn infection is a primary trigger for Aβ-amyloid processing and deposition in brain tissues (Balin et al., 2011). While consistent co-localization of amyloid with *Chlamydia* antigen was not apparent, both were present in the same regions at times consistent with AD-like pathology. As the course of infection preceded the course of pathology development, infection may serve as a stimulus for inflammation as well as for beta amyloid production and deposition. Precedence for infection in exacerbating AD-like pathology has been reported for other types of infections in different animal models (McManus et al., 2014; Wang et al., 2014). Once the infection has been controlled or resolved completely, levels of soluble amyloid apparently decrease presumably following internalization by glial cells (Hawkes et al., 2012) and/or washout into the blood, thereby resulting in fewer deposits documented at the 3 and 4 month timepoints. These findings support our contention that laboratory strains of Cpn from respiratory infections as compared to Cpn brain isolates are less capable of creating long-standing damage in the CNS. In this regard, at the present time, we do not know what inoculum of Cpn is sufficient to not only initiate but to promote chronic human disease, nor do we understand potentially different virulence factors of Cpn isolated from different tissue sites. Our animal studies do support our contention that infection (even in modest titers – 10^5^ organisms), specifically through an intranasal route, can initiate changes in the brain consistent with early AD-like pathology.

In mice infected with the 96-41 Cpn brain isolate, deposits of Aβ-amyloid could be identified as early as 2 months pi, with the greatest numbers at 3 months pi. Furthermore, the increase in both number and size of amyloid deposits suggested the development of progressive AD-like pathology. This is an important issue as early initiating events resulting in sporadic late-onset AD have not been addressed using genetically modified transgenic models that principally emulate familial AD, not the more common late-onset form of disease. Further, experimental animal systems intended to model sporadic late-onset AD have been limited by the absence in understanding specific factors that initiate or promote the early deposition of Aβ-amyloid prior to the onset of symptomatic illness; however, numerous experimentally induced animal models utilizing direct injection of microbial products have been shown to induce transient amyloid production and deposition (Erickson et al., 2012; Krstic et al., 2012). Our current study with a respiratory isolate of Cpn supports the induction of transient amyloid deposition and contrasts with our previous work suggesting that a brain isolate of Cpn results in progressive amyloid accumulation.

Interestingly, a previous study did not identify substantial AD-like pathology in the brain using the respiratory isolate TWAR 2043, a commonly studied laboratory strain of Cpn (Boelen et al., 2007). Boelen et al. (2007) infected BALB/c mice intranasally, and at one and 3 months pi examined brain tissue on the assumption that, both, TWAR 2043 and the AD brain isolate 96-41 reported by Little et al. (2004) would induce similar patterns of pathology following infection. Amyloid beta deposits were reported as 1 or 2 aggregates per section without a preference for a certain brain region in the Boelen et al. (2007) study, and the researchers indicated that Cpn was undetectable in the CNS at 1 or 3 months pi. Both mock-infected and Cpn*-*infected mice exhibited no difference in the number of amyloid deposits. The clear difference noted from the Little et al. (2004) study of total number and size of deposits was notably different from the Boelen et al. (2007) report. It was proposed by Boelen et al. (2007) that discrepancies between reports could have arisen because the TWAR 2043 Cpn strain may have different virulence properties than the 96-41 AD-brain isolate (Balin et al., 2011). It has been observed and we concur that isolates of Cpn propagated long-term in culture may differ substantially from primary respiratory specimens. With respect to the ability to establish a persistent infection and subsequent induction of pathology, TWAR 2043 and 96-41 appear to display different phenotypes in the brains of BALB/c mice.

Our current findings support the contention that isolates of Cpn may differ in their ability to establish chronic or persistent infection and promote progressive pathology. Numerous questions remain as to the nature of the organisms that typically infect the human population. Pertinent issues, just to name a few, include: risk factors promoting infection at specific sites in the body, spread into different tissues and organs following initial infection, virulence factors expressed by the organism and/or host response, and age at which infection occurs. With regards to age, a prior study of Cpn infection in older animals suggests that older age at time of infection promotes the establishment of a brain infection (Little et al., 2005). Further, to address our current study that a modest inoculum of a respiratory isolate of Cpn initiated specific but non-sustainable change in the brain following intranasal inoculation, we preliminarily inoculated a small group of animals with Cpn AR-39, either twice (days 0 and 30), or three times (days 0, 30, and 60) and sacrificed at day 90, and found that individual BALB/c mice inoculated twice displayed 68 amyloid deposits per mouse and those inoculated three times had 177 amyloid deposits per mouse (unpublished observations). In comparison, mice receiving only a single intranasal inoculation as observed in the current study at 3 months pi had an average of 17–18 deposits per mouse (53 deposits/3 mice; see **Table 1A**). These preliminary observations would suggest that multiple inocula of Cpn may exacerbate pathology in the brain, but further experiments are required to clarify this. Furthermore, respiratory or blood-borne organisms may become altered after invading different tissue sites including the brain and this may reflect biovar and serovar differences with Cpn, although this remains to be determined. Future sequencing analyses and specific characterization of different tissue and organ isolates may help to resolve these issues.

In summary, Cpn appears to be a primary trigger for the generation and extracellular deposition of Aβ-amyloid in our mouse model. The differences in progressive as compared to non-progressive pathology may be due to strain variations. This implies that virulence factors and tissue tropism of the organism as well as numerous host factors all contribute to determine the overall pathogenicity of different isolates of Cpn in particular tissues following intranasal infection and subsequent dissemination (Balin et al., 2011). Thus, in concert, host and pathogen interactions initiate detrimental processes that, if left unchecked, may progress to the disease state.

## AUTHOR CONTRIBUTIONS

Christopher S. Little contributed to the conception and design of the work as well as the acquisition, analysis and interpretation of the data and drafting the manuscript, Timothy A. Joyce contributed to the acquisition, analysis and interpretation of the data, Christine J. Hammond contributed to design of the work as well as the acquisition, analysis and interpretation of the data and editing the manuscript, Hazem Matta contributed to the acquisition and interpretation of the data, Denah M. Appelt contributed to the conception of the work as well the interpretation of the data and editing the manuscript, Brian J. Balin contributed to the conception and design of the work as well as the analysis and interpretation of the data and drafting the manuscript.

## Conflict of Interest Statement

The authors declare that the research was conducted in the absence of any commercial or financial relationships that could be construed as a potential conflict of interest.
